# The complete mitochondrial genome of Helan Mountain chukar *Alectoris chukar potanini* (Galliformes: Phasianidae)

**DOI:** 10.1080/23802359.2019.1637792

**Published:** 2019-07-12

**Authors:** Hui Gao, Zhensheng Liu, Yujiao Sun, Chang Zhao, Jifei Wang, Liwei Teng

**Affiliations:** a College of Wildlife Resources, Northeast Forestry University, Harbin, China;; b Key Laboratory of Conservation Biology, National Forestry and Grassland Administration, Harbin, China;; c College of Food and Biological Engineering, Henan University of Animal Husbandry and Economy, Zhengzhou, China;; d Personnel Department, East China University of Science and Technology, Shanghai, China;; e Administration of Helan Mountain National Nature Reserve in Ningxia, Yinchuan, China

**Keywords:** *Alectoris chukar potanini*, complete mitochondrial genome, protein-coding genes, phylogenetic tree

## Abstract

The complete mitochondrial genome of Helan Mountain chukar (*Alectoris chukar potanini*) was first determined. The genome was 16,685 bp in length, comprising 13 protein-coding genes, 22 tRNA genes, two rRNA genes, and a putative control region. The phylogenetic analysis based on the complete mitochondrial genome sequences revealed a close relationship between *A. chukar potanini* and *Tetraogallus tibetanus*. This work is expected to provide a set of fundamental data on further genetic studies of this protected species.

The Helan Mountain chukar, *Alectoris chukar potanini*, is one of the subspecies of *Alectoris chukar* with a limited distribution in northwest of China, mainly in the Helan Mountain area (Liu 1984) at altitudes of 1400–1600 m. In recent years, the population and habitat of *A. chukar potanini* are declining due to the human activities, thus effective measures are badly needed to protect this species. However, little is known about this pheasant, especially the molecular evolution. To better understand and protect *A. chukar potanini*, we first measured and analyzed the complete mitochondrial genome of this species. The tissue specimen was collected from an individual of accidental death in Inner Mongolia Helan Mountain National Nature Reserve, China. The specimen (No. HLSJ201711) was stored in Animal Herbarium of Northeast Forestry University. The genomic DNA was extracted using standard techniques including cell lysis with proteinase K (Sigma, United States) and phenol/chloroform method. Thirteen primer pairs were designed to amplify the complete mitochondrial genome of *A. chukar potanini*.

The total length of the mitogenome (GenBank accession number KT806484) was 16,685 bp, with a base composition of 30.4% A, 24.5% T, 31.6% C, and 13.5% G. The mitogenome contained 13 protein-coding genes, 2 ribosomal RNA (rRNA) genes, 22 transfer RNA (tRNA) genes, and a control region (D-Loop), identical to the typical vertebrate mtDNA. The total length of the 13 protein-coding genes was 11,196 bp, of which 12 genes started with an ATG codon, while *COI* gene began with GTG. In addition, *ND1*, *COII*, *ATP8*, *ATP6*, *ND3*, *ND4L*, *DN5*, and *Cytb* genes ended with the common TAA termination codon, and the *COI* and *ND6* genes stopped with AGG and TAG, respectively, while *ND2*, *COIII*, *ND4* genes ended with an incomplete stop codon ‘T––’. The D-loop region was located between the *tRNA^Glu^* and *tRNA^Phe^* genes, consistent with that of other pheasants such as *Crossoptilon* and *Pucrasia* (Huang and Ke 2015; Li et al. 2015).

To ascertain the taxonomic position of the Helan Mountain chukar, 26 mitogenomes of Phasianidae were retrieved from GenBank and used to construct the phylogenetic tree with three species of genus *Grus* as outgroups (Figure 1). The Maximum likelihood (ML) phylogenetic analysis was conducted in MEGA7 (Kumar et al. 2016) under the GTR + I + G model. The result showed that the subfamily Phasianidae was not monophyletic (Figure 1), congruent with previous studies (Kimball and Braun 2008; Zhao et al. 2012). The *A. chukar potanini* was well grouped with *Tetraogallus tibetanus*, and then both of them were well clustered with *Coturnix chinensis*.

**Figure 1.**
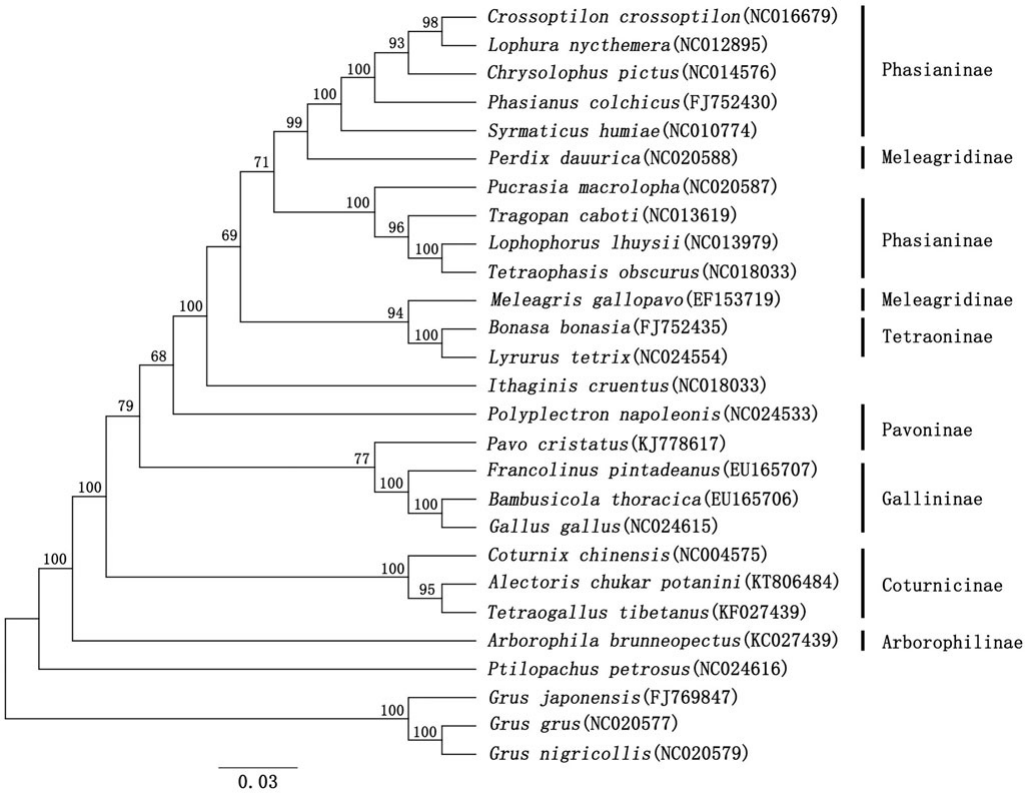
Maximum-likelihood (ML) tree inferred from the complete mitogenomes with the GTR + I + G model in MEGA7 software. The numbers above the lines or beside the nodes represent Bootstrap support (BP). The GenBank accession numbers are given in parentheses.
